# Effects of fig and pineapple powder on metabolite compounds and health-promoting properties in fermented goat meat sausage

**DOI:** 10.5713/ab.24.0526

**Published:** 2024-10-25

**Authors:** Shine Htet Aung, Rupasinghe Ramesh Nimantha, Young-Sun Choi, Aera Jang, Jun Heon Lee, Ki-Chang Nam

**Affiliations:** 1 Department of Animal Science and Technology, Sunchon National University, Suncheon 57922, Korea; 2 Department of Zoology, Kyaukse University, Kyaukse 05151, Myanmar; 3 Livestock Research Institute, Gangin 59213, Korea; 4 Department of Applied Animal Science, College of Animal Life Science, Kangwon National University, Chuncheon 24341, Korea; 5 Division of Animal and Dairy Science, Chungnam National University, Daejeon 34134, Korea

**Keywords:** Biological Activities, Fermented Sausage, Fig, Goat Meat, Metabolite Compounds, Pineapple

## Abstract

**Objective:**

The objective of this research was to identify the metabolite compounds and health-promoting properties of goat meat fermented sausages containing different amounts of fig and pineapple powder (0.1%, 0.25%, and 0.5%), which are presented by F1, F2, F3 and P1, P2, P3, separately.

**Methods:**

Fermented sausages were manufactured from the lean meat of female goats. The samples extracted from the goat meat fermented sausages were evaluated for their metabolite compounds, antioxidants, and angiotensin-converting enzyme inhibitory activity.

**Results:**

The results showed that the diphenyl picrylhydrazyl radical scavenging activity, azino-bis (3-ethylbenzothiazoline-6-sulfonic acid) radical scavenging activity, and angiotensin-converting enzyme inhibitory activity were the highest in F3 (p<0.05), whereas the hydroxyl radical scavenging capacity was the highest in P3 (p<0.05). In addition, F3 and F2 showed the highest superoxide anion radical scavenging activity (p<0.05), whereas F3 and P3 showed the highest copper chelating activity (p<0.05). Based on the metabolite compounds, hydrophobic amino acids, aromatic amino acids, and bitter amino acids were abundant in F3. Both F3 and P3 contained high concentrations of umami-producing compounds.

**Conclusion:**

The incorporation of 0.5% fig powder into goat meat fermented sausage showed improved biological activities based on metabolite compounds.

## INTRODUCTION

In recent years, consumer requirements for safe and healthier foods with consistent eating quality, diversity, and convenience have increased. Therefore, researchers are increasingly focusing on diet-related nutrition to improve health, in addition to investigating medicines. In particular, bioactive peptides have received increasing interest because of their potential nutritional and health benefits. Bioactive peptides are embedded within the primary structure of their parent proteins [1]. However, these peptides are released through proteolysis and become active and capable of exerting physiological effects.

Proteolysis is one of the most significant biochemical alterations occurring during sausage fermentation. Muscle peptidases, including cathepsins, are crucial enzymes involved in the initiation and progression of proteolysis during the processing of meat products, such as fermented sausages [2]. In addition, marination or seasoning contains exogenous proteolytic enzymes such as papain and bromelain, which can further break down proteins in meat [3]. The proteolytic enzyme bromelain, found in pineapples (*Ananas comosus*), aids in digestion by degrading food proteins [4]. The fig (*Ficus carica*), rich in ficin and highly nutritious, is a common tool for tenderizing meat [5]. Therefore, naturally occurring exogenous enzymes like those in fig and pineapple may enhance the proteolysis during fermentation. The proteolysis of proteins during sausage fermentation produces bioactive peptides with many beneficial health effects.

The amino acids and bioactive peptides of varying sizes produced contribute to a range of functionalities and other features, in addition to improving the texture and flavor of meat products. Peptides can perform various biological activities based on their amino acid sequences, including angiotensin-converting enzyme (ACE) inhibitory, anti-hypertension, immune response modulation, blood clot prevention, anti-cancer, and antioxidant activities [6]. Peptides possessing antioxidant activity employ two primary mechanisms to neutralize free radicals: hydrogen atom transfer (HAT) and single electron transfer (SET) [7]. Diphenyl picrylhydrazyl (DPPH) radical scavenging activity is regulated by the HAT mechanism, wherein antioxidant peptides donate a hydrogen atom to free radicals. Higher antioxidant and ACE-I inhibitory activities have been observed in fermented sausages inoculated with lactic acid bacteria (LAB) [8]. Gallego et al [9] reported high ACE inhibitory activity and diphenyl picrylhydrazyl (DPPH) radical scavenging activity in dry fermented sausages from Spain and Belgium. According to Kong et al [10], the observed bioactivity can be attributed to the hydrophobic amino acid-containing peptides found in meat products. Therefore, the health concerns of customers can be addressed by analyzing the potential biological activity of meat products.

In addition, the analysis of metabolic compounds in meat products can offer important information about their safety, flavor, composition, and possible health effects. Metabolites are typically less than 1 kDa in size, represent the end products of the metabolism, and provide a holistic view of physiological states [11]. In the context of fermentation, proteases and lipases produced by LAB play a crucial role in breaking down macromolecules in sausages, producing metabolites that enhance flavor and improve the overall quality of the fermented food [12]. Meat metabolic profiles are associated with sensory appeal, color, oxidative stability, flavor, and aroma [13]. Therefore, these investigations can ensure the safety and quality of fermented sausages. However, only a few studies have been conducted to determine the metabolic components of fermented sausages containing fig and pineapple powders.

Consequently, a lot of attention has been placed on researching the biological activity of meat products and developing goods that enhance both human health and their quality attributes. Therefore, this research aimed to evaluate the antioxidant and ACE-I inhibitory properties of the goat meat fermented sausage extract combined with fig and pineapple powders. Moreover, we aimed to compare the metabolite compounds in different fermented sausages.

## MATERIALS AND METHODS

### Preparation of fermented sausage

The lean meat was kept for further use at 4°C after trimming all visible connective tissue and fat from female goats’ *M. biceps femoris* and *M. semitendinosus*. The formulations used in fermented sausage manufacturing are listed in Table 1. Seven different sausage types were prepared using different concentrations of pineapple and fig powders (control, 0.1%, 0.25%, and 0.5%) according to the preliminary trial. Pineapple powder and fig powder (100% pure) were obtained from Saemaeul, a food additive company based in Changnyeong, Korea. The carbohydrates, protein, and fat content of pineapple powder (79.9%, 2.95%, and 0.96%) whereas fig powder (81%, 4%, and 4%) respectively.

**Figure 1:**
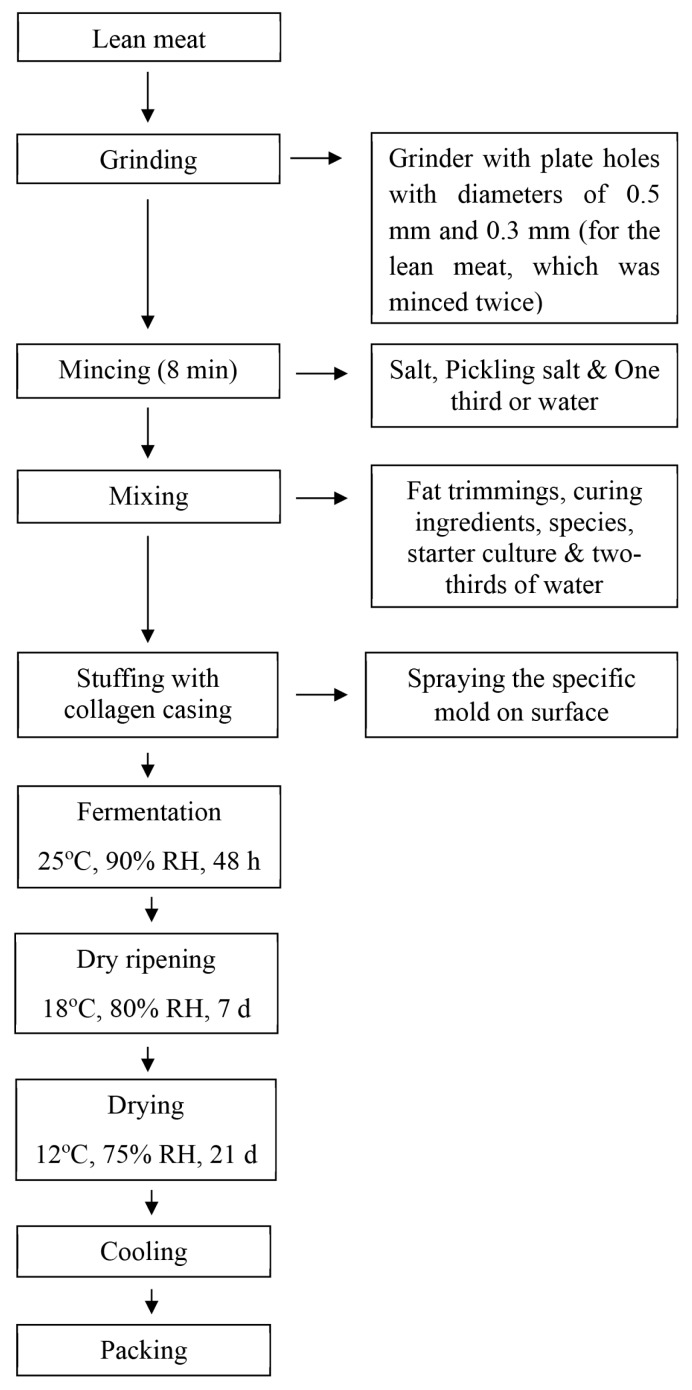
The diagram for the manufacturing process of goat meat fermented sausage. RH, relative humidity.

Figure 1 illustrates the sausage manufacturing process. The sausages were processed at three different levels (fermentation, dry ripening, and drying) in a dough conditioner (Grand Woosung, Seoul, Korea) for 30 d. The fermented sausages were cooled and packaged for further analysis.

### Extraction obtained from the goat meat fermented sausage

The method of Kim et al [14] was used to extract proteins from the goat meat fermented sausages with minor modifications. The sample (2 g) was mixed with 10 mL of 0.02M phosphate buffer (pH 7.4) and homogenized for 30 s at 3,700×g. After centrifuging the homogenate at 1,500×g for 15 min at 4°C, the supernatant was collected. Afterwards, a freeze dryer (Lyoph-Pride, LP03; Ilshin BioBase Co., Ltd., Dongducheon, Korea) was used to lyophilize the samples to investigate their functional activity.

### Measurement of functional activities

#### Diphenyl picrylhydrazyl radical scavenging activity

The antioxidant activity of goat meat fermented sausage peptides was evaluated using the DPPH radical scavenging activity measurement procedure [15]. The extracted samples were diluted in 50 volumes of distilled water (DW). After mixing 2 mL of the diluted sample solution with 18 mL of DW, centrifugation was performed for 10 min at 1,500×g. Afterwards, 0.4 mL of supernatant was placed in a 50 mL centrifuge tube with 1.6 mL DW and 2 mL DPPH (0.2 mM in methanol). After storing the mixture in total darkness for 1 h, centrifugation was performed for 10 min at 1,500×g. The following formula was used to assess antioxidant activity after absorbance was measured at 517 nm.


$$\text{DPPH}-\text{radical scavenging activity }(\%)=\frac{\text{Absorbance of control}-\text{Absorbance of sample}}{\text{Absorbance of control}}\times 100$$

### Superoxide anion radicals scavenging activity (%)

The procedure published by Gao et al [16] was used to investigate the superoxide anion scavenging capacity of the goat meat fermented sausage peptides, with minor modifications [17]. In short, test 2 was carried out by first mixing 0.8 mL of the sample solution (0.1 mg/mL) with 0.2 mL of Tris-HCl buffer (0.1 M, pH 8.0). The absorbance at 320 nm was then measured. After this initial measurement, 0.1 mL of a 3 mM pyrogallol solution was added to the mixture, and the absorbance at 320 nm was measured again as test 1. The activity was calculated as a percentage using the formula below:


$$\text{Resistance to superoxide anion radicals }(O.\bar{2})(\%)=\left[1-({\text{A}}_{\text{test}\;1}-{\text{A}}_{\text{test}\;2)}/{\text{A}}_{\text{test blank}}\right]\times 100$$

where: A_test 1_: goat meat fermented sausage extract with pyrogallol, A_test 2_: goat meat fermented sausage extract without pyrogallol, and A_test blank_: DW with pyrogallol.

### Hydroxyl radical scavenging capacity (%)

The hydroxyl radicals (·OH) scavenging activity was assessed by Shi et al [17]. In short, 1 mL of goat meat fermented sausage peptides (0.1 mg/mL) was mixed with 1 mL 1,10-phenanthroline (0.75 mM), 1.5 mL sodium phosphate buffer (0.15 M, pH 7.4), 1 mL FeSO_4_ (0.75 mM), and 1 mL H2O2 (0.01 %, v/v). Subsequently, the solution was heated for 30 min at 37°C. The change in absorbance at 536 nm was then measured using a spectrophotometer, which indicated the ability to scavenge hydroxyl radicals.


$$\text{Resistance of hydroxyl radical }(\%)=({\text{A}}_{\text{test}}-{\text{A}}_{\text{blank}})/({\text{A}}^{\prime }-{\text{A}}_{\text{blank}})\times 100$$

where: *A_test_*: solution with goat meat fermented sausage peptides; *A′*: solution where goat meat fermented sausage peptides and H_2_O_2_ were replaced with DW; *A_blank_*: blank sample where goat meat fermented sausage peptides were replaced with DW.

### Azinobis(3-ethylbenzothiazoline-6-sulfonic acid)

The Azinobis(3-ethylbenzothiazoline-6-sulfonic acid) (ABTS^+^) radical scavenging activity was investigated using the procedure reported by Shi et al [17]. The lyophilized goat meat fermented sausage extract was re-dissolved (0.1 mg/mL) concentration in DW. The radical cation ABTS^+^ solution was made through the amalgamation of 7 mM ABTS and 2.45 mM potassium persulfate. Subsequently, it underwent heating at a standard temperature in absolute darkness for 15 h. The ABTS^+^ solution underwent dilution with methanol until it achieved a meticulously controlled absorbance range of 0.700±0.020 at the specific wavelength of 734 nm. The absorbance was then determined at 734 nm after the test sample solution (100 μL) was combined with the ABTS+ solution (3 mL). A blank sample was prepared using distilled were instead of the sample solution. ABTS^+^ radical scavenging activity was calculated using the equation below:


$${\text{ABTS}}^{+}\;\text{scavenging activity }(\%)=\left(1-\frac{{\text{A}}_{\text{test}}}{{\text{A}}_{\text{blank}}}\right)\times 100$$

where: *A_test_*: solution with goat meat fermented sausage peptides and *A_blank_*: a blank sample was prepared with distilled water. The inhibitory concentration at 50% (IC_50_) value was used to represent the scavenging activity.

### Copper (II) chelating activity

The assessment of copper (II) chelating activity was conducted following the procedure delineated by Kong and Xiong [18]. In a 15 mL tube, 1 mL of the test sample solution was mixed with an equal volume of 0.2 mM copper (II) sulfate solution. The resultant mixture was left to react for a duration of 5 min at room temperature. The solution was then treated with an 11.3% trichloroacetic acid (TCA) solution (1 mL) prior to being centrifuged at 2,500×g for 10 min. Next, the supernatant (2 mL) was subjected to a treatment of 1 mL of 10% pyridine and 20 μL of 0.1% pyrocatechol violet in a centrifuge tube. The mixture underwent a further incubation of five minutes at room temperature. Subsequently, the supernatant was collected after centrifugation at 2,500×g for 10 min. The absorbance of the reaction supernatant was then measured at 632 nm.


$$\text{Copper }(\text{II})\;\text{chelating activity}=1-\left(\frac{\text{Absorbance of sample}}{\text{Absorbance of blank}}\right)\times 100$$

Copper (II) chelating activity was expressed with IC_50_ value.

### Angiotensin-converting enzyme-I converting enzyme inhibitory activity

A few modifications were made to the ACE inhibitory activity of goat meat fermented sausage peptides based on earlier research [19]. The test sample solution (100 μL) was treated with 100 μL of borate buffer (0.1 M, pH 8.3), which was mixed with 5 mM Hippuryl-Histidil-Leucine, 20 μL of ACE (0.25 U), and 0.3 mM NaCl. Subsequently, the mixture received 150 μL of HCL (1 M) treatment and was heated for 30 min at 37°C. Centrifugation was performed at 1,500×g for 10 min after adding 1 mL of ethyl acetate to extract the produced hippuric acid, and. The supernatant (750 μL) was then placed in a glass tube and a heat blot was used to evaporate it for 15 min at 90°C. The hippuric acid was then again dissolved using 800 uL of DW, and absorbance was measured at 228 nm with a microcuvette. A blank sample without the test sample solution was prepared.


$$\text{ACE inhibition }(\%)=\frac{\left({\text{A}}_{\text{blank}}-{\text{A}}_{\text{sample}}\right)}{{\text{A}}_{\text{blank}}}\times 100$$

ACE inhibition was expressed with IC_50_ value.

### Nuclear magnetic resonance spectroscopy

Sample extraction procedures were executed in accordance with the methodologies detailed by Kim et al [20]. Initially, each treatment sample underwent extraction with 20 mL of 0.6 M perchloric acid. Following homogenization, the resulting homogenate was centrifuged for 20 min at 3,500×g using a Continent 512R centrifuge (Hanil, Daejeon, Korea). Subsequently, the supernatant was subjected to another round of centrifugation under identical conditions after treatment with a KOH solution to adjust the pH to 7.0. Subsequently, the samples were filtered through a Whatman No.1 filter paper and lyophilized (Lyoph-Pride, LP03; Ilshin BioBase). The lyophilized material was then diluted in 1 mL of D_2_O containing 1 mM 3-(trimethylsilyl)propionic-2,2,3,3-d4 acid and pH 7.4 adjusted with 20 mM phosphate buffer (1 mM TSP buffer) and allowed to stand for roughly 10 min at 38°C in the water bath. Following the initial centrifugation at 3,500×g for 10 min, the supernatant was transferred to a microcentrifuge tube. Subsequently, a secondary centrifugation step at 12,000×g for 10 min was employed to further refine the separation of components. The supernatant, comprising 600 μL, was carefully transferred to an nuclear magnetic resonance spectroscopy (NMR) tube for subsequent analysis. Employing a Bruker 600 MHz cryo-NMR spectrometer (Bruker Biospin, Rheinstetten, Germany), the NMR analysis was conducted. Subsequently, Topspin 4.0.8 (Bruker Biospin) software was utilized for spectral analysis, facilitating quantitative assessment with TSP serving as a reference.

### Statistical analysis

Experimental data with three replicates of each treatment were analyzed using R statistical programming (version: 4.3.2). The data were statistically analyzed using one-way analysis of variance at a 95% confidence level. The statistically significant variations between mean values were ascertained using Tukey’s multiple range test. MetaboAnalyst 6.0 (https://www.metaboanalyst.ca/) was used to compute the partial least squares discriminant (PLS-DA) and variable importance projection (VIP) scores, and the TB tool was used to compute the heatmap.

## RESULTS AND DISCUSSION

### Diphenyl picrylhydrazyl radical scavenging activity

**Figure 2:**
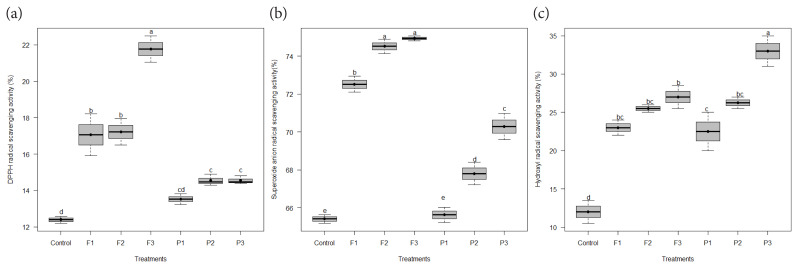
Results of (a) DPPH radical scavenging activity (%), (b) superoxide anion radical scavenging activity (%), (c) Hydroxyl radical scavenging activity (%) of the extract of goat meat fermented sausages incorporating exogenous enzymes. Treatments: Control, without powder; P1, 0.1% pineapple powder; P2, 0.25% pineapple powder; P3, 0.5% pineapple powder; F1, 0.1% fig powder; F2, 0.25% fig powder; F3, 0.5% fig powder. DPPH, diphenyl picrylhydrazyl. ^a–e^ Mean values within boxplot with different superscripts differ significantly (p<0.05).

The incorporation of exogenous enzymes into goat meat fermented sausage had a substantial effect on DPPH radical scavenging activity (Figure 2a). The control group showed the lowest DPPH radical scavenging activity, while F3 had the highest (p<0.05). F1 and F2 were lower than F3 but higher than other treatments (p<0.05). There were no significant differences among P1, P2, and P3, and P1 did not differ from the control (p>0.05). According to these results, fig powder incorporated in goat meat fermented sausages had higher DPPH radical scavenging activity than pineapple powder. This result may be due to differences in the peptides released from goat meat fermented sausages, such as molecular weight, amino acid sequence, and hydrophobic or hydrophilic characteristics. According to Esfandi et al [7], tyrosine-containing peptides often function as antioxidants via the HAT pathway. In addition, peptide fractions exhibiting higher levels of hydrophobic aliphatic (valine, isoleucine, and leucine) and aromatic (phenylalanine and tyrosine) amino acid residues showed higher DPPH radical scavenging activity [21]. The most well-known antioxidant peptides found in meat proteins are carnosine and anserine. In the present study, F3 had the highest levels of isoleucine, leucine, valine, anserine, tyrosine, and carnosine, all of which respond to oxidative stress by suppressing radicals.

### Superoxide anion radicals scavenging activity

Reactive oxygen species (ROS), including superoxide anion (***O***.2̄), hydrogen peroxide (H_2_O_2_), hydroxyl radical (·OH), and others are highly reactive molecules containing oxygen [22]. When meat is exposed to oxygen during storage, handling, and processing, there is a potential for the generation of superoxide anions through oxidative reactions involving lipids and proteins. In response to the presence of superoxide anions, endogenous meat enzymes and small molecule antioxidants scavenge superoxide anions, thereby protecting meat proteins from oxidative damage. Recently, F3 and F2 had the highest scavenging activity, followed by F1, P3, P2, P1, and the control (p<0.05) (Figure 2b). However, there were no significant differences between the control and P1 (p>0.05). The incorporation of fig powder into goat meat fermented sausages had a strong effect on (***O***.2̄)-scavenging activity. The presence of aromatic amino acids (tryptophan and phenylalanine) in peptides significantly contributes to their radical-scavenging properties by providing proton-donating capabilities, which help counteract the oxidative damage caused by free radicals [23]. Similar findings were reported by Ren et al [24], it was observed that tryptophan and phenylalanine, two aromatic amino acids, were associated with their capability to serve as radical scavengers. Preedy [25] demonstrated the antioxidant action of carnosine by scavenging peroxyl and superoxide free radicals. Phenylalanine was highest in F3 and F2, whereas carnosine was the highest in F3 of the current result.

### Hydroxyl radical scavenging capacity

Highly ROS include the hydroxyl radical (·OH). This radical reacts with a wide range of molecules in biological systems, including proteins, lipids, and DNA, leading to damage and potentially harmful consequences. However, peptides can also have hydroxyl radical (·OH) scavenging capacity. Recent findings indicate that peptides originating from P3 exhibit the highest capacity to scavenge hydroxyl radicals (p<0.05), whereas the control displayed the lowest scavenging activity (p<0.05) (Figure 2c). No significant differences were observed among F3, P2, F2, and F1 (p>0.05). In addition, P1 did not significantly differ from those of P2, F2, and F1 (p>0.05) but was higher than the control and lower than F3 (p<0.05). Escudero et al [26] have reported that histidine-enriched peptides have been recognized for the potential to serve as radical scavengers because they can chelate metal ions, scavenge hydroxyl radicals, and operate as active oxygen quenchers. Metal chelating activity is crucial for the inhibition of hydroxyl radicals because Fe^2+^ is oxidized to Fe^3+^ by hydrogen peroxide, forming hydroxyl radicals and hydroxide ions. In addition, the reaction of ·OH can be inhibited by ·OH scavengers, such as glucose, which can act as precursors for the synthesis of certain antioxidant molecules, such as ascorbic acid (vitamin C) and glutathione, which can scavenge hydroxyl radicals [27]. According to the current results, histidine was the most prevalent in F3, whereas glucose was the most prevalent in F3 and P3.

### Azinobis(3-ethylbenzothiazoline-6-sulfonic acid) radical scavenging activity

**Figure 3:**
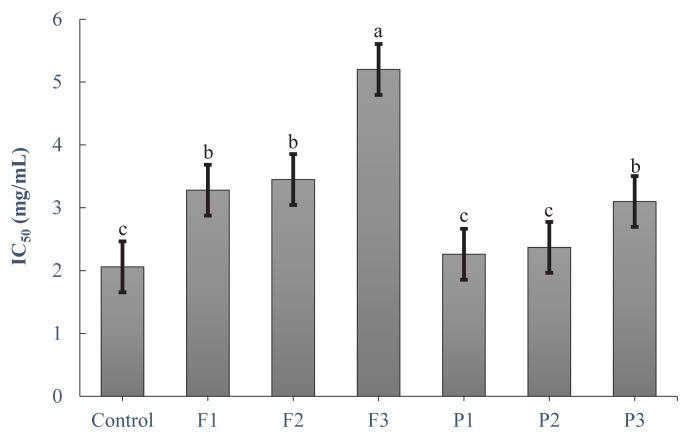
ABTS^+^ radical scavenging activity (IC_50_) of the extract of goat meat fermented sausages incorporating exogenous enzymes. Treatments: Control, without powder; P1, 0.1% pineapple powder; P2, 0.25% pineapple powder; P3, 0.5% pineapple powder; F1, 0.1% fig powder; F2, 0.25% fig powder; F3, 0.5% fig powder. ^a–c^ Mean values within the bar chart with different superscripts differ significantly (p<0.05).

ABTS^+^ radical scavenging activity showed a similar trend to that of the DPPH radical scavenging activity (Figure 3). F3 had the highest scavenging activity (p<0.05), whereas P1, P2, and the control showed the lowest activity (p<0.05). F1, F2, and P3 were significantly lower than F3 and higher than P1, P2, and the control (p<0.05). SET mechanism controls the activity of the ABTS^+^ radical scavenging system by providing electrons for the transformation of free radicals into radical cations. Peptides comprising cysteine, histidine, and tryptophan participate in several biological activities via the SET mechanism [7]. F3 had a greater histidine level in the current investigation.

### Copper (II) chelating activity

**Figure 4:**
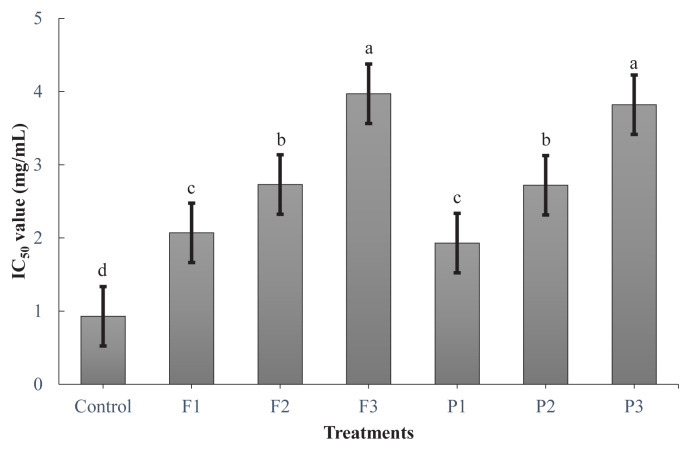
Copper chelating activity (IC_50_) of the extract of goat meat fermented sausages incorporating exogenous enzymes. Treatments: Control, without powder; P1, 0.1% pineapple powder; P2, 0.25% pineapple powder; P3, 0.5% pineapple powder; F1, 0.1% fig powder; F2, 0.25% fig powder; F3, 0.5% fig powder. ^a–d^ Mean values within the bar chart with different superscripts differ significantly (p<0.05).

Transition metals, such as iron and copper, can indeed catalyze the reduction of hydroperoxides to reactive radical species through a metal-catalyzed oxidation mechanism [28]. Copper has a higher redox potential than iron, which means that it can undergo oxidation and reduction reactions more readily, making it more efficient in catalyzing the decomposition of hydroperoxides. Antioxidants or metal chelators break the chain reaction by reacting with radicals and stopping it from spreading further. In the current study, peptides generated from goat meat fermented sausages containing exogenous enzymes showed high metal-chelating activity (Figure 4). F3 and P3 showed the highest copper-chelating activities (p<0.05), whereas the control showed the lowest activity (p<0.05). Incorporating higher concentrations of exogenous enzymes (fig and pineapple powders) into goat meat fermented sausages resulted in a significantly higher copper-chelating activity. This could be attributed to several factors inherent to the fermentation process and the nature of peptides generated during fermentation. The rationale for this is that amino acid residues, including aspartate, histidine, glutamate, phosphorylated serine and threonine, can bind metals [28]. According to previous studies, histidine-containing peptides can act as metal chelators through coordination bonds formed between the nitrogen atoms of the imidazole group in histidine and the metal ions [23]. Furthermore, Lee et al [29] have reported that methionine, an amino acid containing sulfur, tends to improve the metal chelation activity. In the current study, amino acids including glutamate, methionine, and histidine were more abundant in F3, whereas aspartate and threonine were higher in P3.

### Angiotensin-converting enzyme-I inhibitory activity

**Figure 5:**
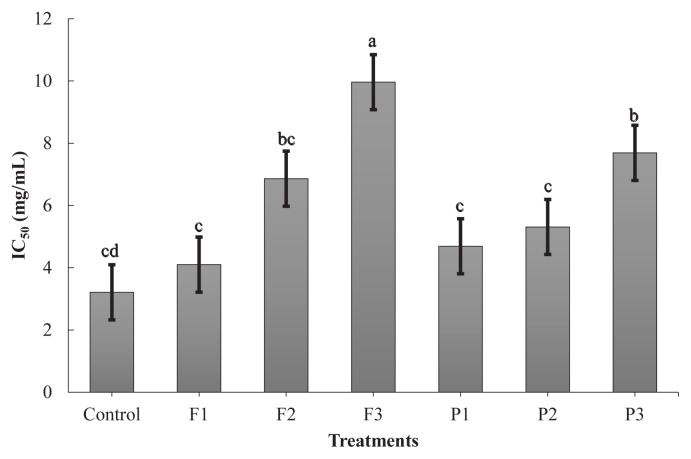
ACE inhibitory activity (IC_50_) of the extract of goat meat fermented sausages incorporating exogenous enzymes. Treatments: Control, without powder; P1, 0.1% pineapple powder; P2, 0.25% pineapple powder; P3, 0.5% pineapple powder; F1, 0.1% fig powder; F2, 0.25% fig powder; F3, 0.5% fig powder. ^a–d^ Mean values within the bar chart with different superscripts differ significantly (p<0.05).

In addition to its antioxidant activity, its ACE-I inhibitory activity has been thoroughly investigated as a functional property because it is responsible for hypertension. ACE converts the inert decapeptide angiotensin I into the effective vasoconstrictor octapeptide angiotensin II. Therefore, this enzyme reaction considerably contributes to the constriction of blood vessels, raising blood pressure [30]. Furthermore, ACE inhibits the activity of bradykinin, a vasodilator. However, ACE inhibitor peptides can block the activity of ACE-I, that are involved in regulating blood pressure. Meat and meat proteins can release ACE inhibitor peptides [31], because the process of proteolysis, facilitated by endogenous or exogenous enzymes, contributes to an increase in the potent ACE inhibitory activity by generating small peptides with unspecified cleavage sites [32]. According to the current study, the fig and pineapple powder-treated goat meat fermented sausages showed a good ability to inhibit ACE activity (Figure 5). There was no significant difference among the control, F1, F2, P1, and P2 (p>0.05). Furthermore, F2 was not significantly different from P3, according to the statistics (p>0.05). In particular, F3 demonstrated the strongest ACE inhibitory activity (p<0.05), followed by P3. It is possible due to certain components of figs that may contribute to the management of hypertension. Potassium, which is found in figs, is a mineral that balances the effects of sodium and is essential for controlling blood pressure [33]. Additionally, different peptide fractions, amino acid sequences, and amino acid residues have different inhibitory abilities. According to De Leo et al [34], peptides with high concentrations of hydrophobic amino acids, aromatics (tyrosine or phenylalanine), or proline at the C-terminus can inhibit the action of ACE. Moreover, Wu et al [35] reported that dipeptides and tripeptides must have specific structural characteristics to inhibit ACE, such as leucine, isoleucine, valine, proline, tryptophan, phenylalanine, and tyrosine at the C-terminus. The results of this study indicate that F3 and P3 had the highest concentrations of aromatic, hydrophobic, and N-terminal aliphatic amino acids, respectively.

### Nuclear magnetic resonance spectroscopy spectroscopy

**Figure 6:**
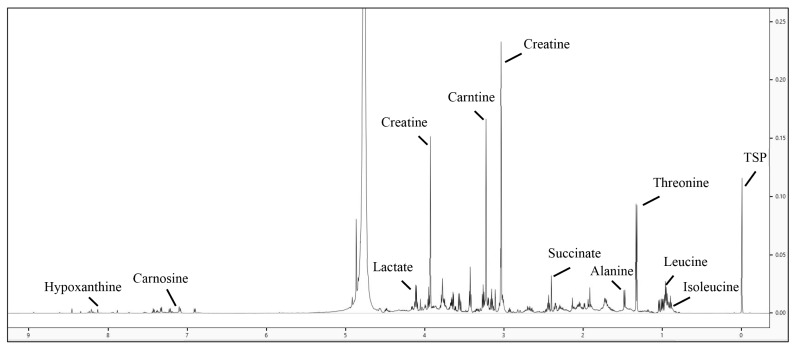
1D^1^H NMR differential concentration spectrum of perchloric acid extract of goat meat fermented sausage. TSP, 3-(trimethylsilyl)propionic- 2,2,3,3-d4 acid

**Figure 7:**
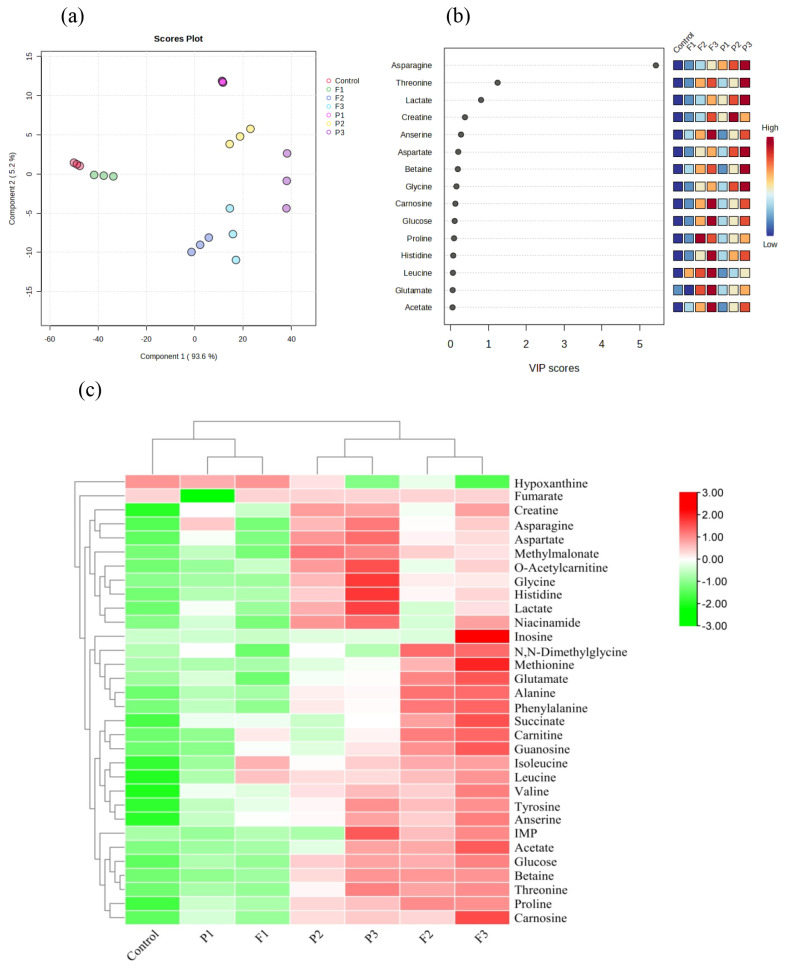
Results of (a) PLS-DA, (b) VIP score, and (c) heatmap for metabolite compounds in goat meat fermented sausage incorporating exogenous enzymes. Treatments: Control, without powder; P1, 0.1% pineapple powder; P2, 0.25% pineapple powder; P3, 0.5% pineapple powder; F1, 0.1% fig powder; F2, 0.25% fig powder; F3, 0.5% fig powder. VIP, variable importance projection; PLS-DA, partial least squares discriminant.

Figure 6 displays the 1D^1^H NMR spectra of goat meat fermented sausage. Various metabolites were observed in the goat meat fermented sausages containing fig and pineapple powders using 1D^1^H NMR spectra. Based on PLS-DA, these metabolites showed significant geographic differences (Component 1, 93.6%) between treatments (Figure 7a). The VIP score reflects the major different compounds for PLS-DA, which included 15 compounds such as asparagine, threonine, lactate, creatine, anserine, aspartate, betaine, glycine, carnosine, glucose, histidine, proline, leucine, glutamate, and acetate (Figure 7b).

Sausages undergo complex biochemical changes during the fermentation process, resulting in the production of various metabolites. Some of the metabolites found in the final products are listed in Table 2. Asparagine, aspartate, betaine, glycine, and threonine were the most abundant amino acids in P3, whereas glutamate, histidine, and leucine were found in F3, and proline was found in F2. Asparagine is identified as one of the tasteless amino acids [36]. Betaine can enhance flavor and is frequently added to meat products to improve their overall quality and sensory aspects. Histidine, leucine, and proline within a peptide sequence contribute to the perception of bitterness [37]. Many bitter dipeptides inhibit ACE. Glutamate, which is produced through proteolysis or glutamine conversion by glutaminase, contribute to the umami taste of fermented foods [38]. Along with glutamate, aspartate plays a significant role in umami perception. Hydrophobic amino acids are rich in antioxidants and ACE inhibitory activities because they can improve their free radicals scavenging efficiency and inhibitory activity [39]. In the current results, hydrophobic amino acids including isoleucine, methionine, and phenylalanine were the most abundant in F3, while alanine, valine, and leucine were the most abundant in F3 and F2 (p<0.05).

Among the bioactive compounds, anserine and carnosine are antioxidant-rich dipeptides with imidazole rings that can donate and receive electrons, eliminate ROS and lessen oxidative stress [40]. The current results showed that anserine and carnosine had the highest levels in F3, whereas anserine and carnitine had no significant difference among F3, P3, and F2 (p>0.05). However, o-acetylcarnitine exhibited the highest abundance within the P3 group, surpassing the levels observed in the other groups with statistical significance (p<0.05). Interestingly, carnosine and anserine have a favorable impact on the organoleptic quality because they are linked to the umami flavor [41].

Five energy metabolism-related compounds were identified in the NMR spectra of the goat meat fermented sausages. Creatine levels were the highest in P2, F3, and P3 (p<0.05), followed by lactate in P3 (p<0.05), and succinate in F3. However, fumarate and glucose levels did not differ significantly between the treatments (p>0.05). Creatine, which is stored in the muscles as creatine phosphate, contributes to the savory or umami flavor of cooked meat by interacting with methylglyoxal. Creatine may have antioxidant properties, potentially reducing oxidative stress in muscle cells. Lactate and succinate are organic acids commonly associated with microbial metabolism, particularly during fermentation [42]. They can contribute to the quality control, safety, and flavor development of fermented meat products.

A comparison of nucleotide-related compounds showed that F3 and F2 had the most guanosine, P3 had inosine monophosphate (IMP) and F3 had inosine, whereas F3 and P3 had the lowest hypoxanthine content (p<0.05). IMP is a nucleotide involved in purine metabolism and a precursor for synthesizing both adenosine and guanosine nucleotides. IMP is indeed known for its contribution to the umami taste of foods [43]. IMP can be dephosphorylated to form inosine, which can be metabolized to hypoxanthine by enzymes. The presence of hypoxanthine in certain foods contributes to a bitter taste. Mesquita Casagrande et al [44] claimed that hypoxanthine is a pro-oxidative cofactor in live animals that produces superoxide anions when combined with oxygen. This may have been related to oxidation.

During fermentation, microorganisms metabolize sugars anaerobically, leading to the production of various metabolic byproducts, including organic acids such as acetate [45]. The flavor and aroma of fermented foods are enhanced by organic acids, which are also crucial for food safety and preservation. Acetate was the highest in F3, P3, and F2 (p<0.05) but the other treatments did not significantly differ from one another (p>0.05). In addition, methylmalonate was the highest in P2 and P3 (p<0.05), followed by F3, F2, P1, F1, and the control. The highest niacinamide levels were observed in P3, P2, and F3 (p<0.05), whereas the other treatments did not significantly differ from one another (p>0.05). Nicotinamide, also known as niacinamide, is a form of vitamin B3 derived from niacin. Nicotinamide is also found in meat products and is produced by the breakdown of pyridine nucleotides [46]. Niacin and its derivatives can indirectly reduce oxidative stress by supporting the enzymatic’ activity to neutralize free radicals [47].

A heatmap was generated for further correlation to compare the differences in metabolite compounds between treatments. The heatmap provides a clear visual representation of the smallest and largest variables within each cluster (Figure 7c). According to the heatmap, F3 had an effect on most metabolite compounds, followed by F2 and P3. According to metabolite compounds, fermented sausages with fig powder had high levels of antioxidant compounds; nevertheless, both sausage types had different flavor-enhancing compounds, where higher powder concentrations tended to have higher activity.

## CONCLUSION

In this study, goat meat fermented sausages incorporated with exogenous enzymes exhibited high functional activity through proteolysis. Especially, the goat meat fermented sausage with 0.5% fig powder (F3) showed better antioxidant and ACE inhibitory activities, followed by the sausage with 0.5% pineapple powder. In the metabolite compounds, the hydrophobic amino acids, aromatic amino residues, and bitter amino acids were abundant in goat meat fermented sausage with 0.5% fig powder (F3). Both 0.5% fig and pineapple powder-treated goat meat fermented sausages (F3 and P3) contained high concentrations of umami-producing compounds. In conclusion, the proteolytic activity of exogenous enzymes during sausage fermentation produces bioactive peptides with functional and health-promoting activities.

## Figures and Tables

**Table 1:** Goat meat fermented sausage manufacturing formulation

Items (%)	Treatments^1)^						

	Control	P1	P2	P3	F1	F2	F3
Goat meat	85	85	85	85	85	85	85
Pork back fat	15	15	15	15	15	15	15
Cold water	16	16	16	16	16	16	16
Sugar	2.5	2.5	2.5	2.5	2.5	2.5	2.5
Salt	1.6	1.6	1.6	1.6	1.6	1.6	1.6
Pickling salt	0.06	0.06	0.06	0.06	0.06	0.06	0.06
Vitamin C	0.05	0.05	0.05	0.05	0.05	0.05	0.05
Starter culture	0.05	0.05	0.05	0.05	0.05	0.05	0.05
Glucose	5	5	5	5	5	5	5
Garlic powder	0.2	0.2	0.2	0.2	0.2	0.2	0.2
Black pepper powder	0.3	0.3	0.3	0.3	0.3	0.3	0.3
Pineapple powder	-	0.1	0.25	0.5	-	-	-
Fig powder	-	-	-	-	0.1	0.25	0.5

1) Control, without powder; P1, 0.1% pineapple powder; P2, 0.25% pineapple powder; P3, 0.5% pineapple powder; F1, 0.1% fig powder; F2, 0.25% fig powder; F3, 0.5% fig powder.

**Table 2:** Results of metabolite compounds (mg/100 g) in goat meat fermented sausage incorporating exogenous enzymes

Compound	Treatments^1)^							SEM

	Control	F1	F2	F3	P1	P2	P3	
Free amino acids								
Alanine	1.29^c^	1.49^c^	2.33^a^	2.36^a^	1.55^c^	1.87^b^	1.84^b^	0.09
Asparagine	46.06^e^	56.01^d^	92.46^c^	105.36^b^	106.23^b^	110.76^b^	127.41^a^	1.90
Aspartate	1.85^c^	2.18^c^	3.54^b^	3.77^b^	3.36^b^	4.47^a^	4.87^a^	0.19
Betaine	3.45^d^	4.17^c^	7.34^a^	7.35^a^	3.95^c^	6.28^b^	7.39^a^	0.17
Glutamate	5.56^cd^	5.16^d^	7.42^a^	7.82^a^	6.06^bc^	6.32^b^	6.41^b^	0.26
Glycine	0.63^e^	0.78^d^	1.80^c^	1.82^c^	0.85^d^	2.25^b^	3.38^a^	0.05
Histidine	2.13^c^	2.45^bc^	2.91^b^	3.93^a^	2.47^bc^	3.13^b^	3.33^b^	0.21
Isoleucine	0.93^d^	2.71^a^	2.76^a^	2.82^a^	1.60^c^	2.24^b^	2.54^ab^	0.12
Leucine	1.36^d^	3.20^b^	3.35^ab^	3.64^a^	2.34^b^	3.13^b^	3.14^b^	0.12
Methionine	0.60^d^	0.60^d^	1.28^b^	1.90^a^	0.62^d^	0.83^cd^	0.93^c^	0.09
N, N- Dimethylglycine	0.05^b^	0.04^b^	0.08^a^	0.08^a^	0.06^b^	0.06^b^	0.05^b^	0.01
Phenylalanine	1.26^e^	1.37^e^	2.57^b^	2.66^a^	1.60^d^	2.01^c^	1.93^c^	0.03
Proline	1.98^c^	2.74^b^	4.15^a^	4.12^a^	2.97^b^	3.62^a^	3.77^a^	0.18
Threonine	3.72^d^	7.25^cd^	26.51^a^	28.67^a^	9.29^c^	18.38^b^	30.02^a^	1.55
Tyrosine	0.86^b^	1.15^a^	1.29^a^	1.36^a^	1.09^a^	1.20^a^	1.31^a^	0.08
Valine	0.88^c^	1.47^b^	1.71^ab^	1.95^a^	1.52^b^	1.66^ab^	1.77^ab^	0.10
Bioactive compounds								
O-Acetylcarnitine	0.40^e^	0.53^cd^	0.58^c^	0.68^b^	0.46^de^	0.76^b^	0.87^a^	0.03
Anserine	8.08^b^	12.84^ab^	13.94^a^	15.75^a^	11.60^ab^	12.98^ab^	14.93^a^	1.25
Carnitine	5.38^c^	7.22^b^	8.44^a^	8.68^a^	5.75^c^	6.39^bc^	7.11^b^	0.32
Carnosine	1.05^e^	1.62^d^	3.06^b^	4.46^a^	2.23^c^	2.99^b^	3.18^b^	0.15
Energy metabolism-related compounds								
Creatine	12.08^c^	15.40^b^	16.24^b^	18.44^a^	16.53^b^	18.50^a^	18.41^a^	0.46
Fumarate	0.01	0.01	0.01	0.01	0.00	0.01	0.01	0.003
Glucose	4.04	4.46	5.90	6.24	4.67	5.66	5.99	0.57
Lactate	2.58^e^	4.32^d^	6.50^c^	9.38^b^	7.92^c^	11.33^b^	15.55^a^	0.39
Succinate	0.49^d^	0.93^c^	1.22^b^	1.43^a^	0.93^c^	0.83^c^	0.97^c^	0.06
Nucleotide related compounds								
Guanosine	0.02^c^	0.07^b^	0.11^a^	0.13^a^	0.03	0.06^b^	0.08^b^	0.004
Hypoxanthine	0.95^a^	0.95^a^	0.90^ab^	0.84^a^	0.94^a^	0.92^a^	0.86^b^	0.01
IMP	1.08^d^	1.12^cd^	1.59^c^	1.77^b^	1.03^d^	1.09^d^	1.93^a^	0.06
Inosine	0.30^b^	0.29^b^	0.35^b^	1.24^a^	0.31^b^	0.36^b^	0.37^b^	0.12
Others								
Acetate	2.19^b^	2.39^b^	3.33^a^	3.75^a^	2.34^b^	2.72^b^	3.37^a^	0.16
Methylmalonate	0.17^d^	0.17^d^	0.27^b^	0.26^b^	0.21^c^	0.32^a^	0.31^a^	0.01
Niacinamide	0.59^b^	0.58^b^	0.67^b^	0.81^a^	0.67^b^	0.82^a^	0.86^a^	0.03

SEM, standard error of the mean.

1) Control, without powder; P1, 0.1% pineapple powder; P2, 0.25% pineapple powder; P3, 0.5% pineapple powder; F1, 0.1% fig powder; F2, 0.25% fig powder; F3, 0.5% fig powder.

a–e Mean values within the same row with different superscripts differ significantly (p<0.05).
